# Clinical Applications of Visual Plasmonic Colorimetric Sensing

**DOI:** 10.3390/s20216214

**Published:** 2020-10-30

**Authors:** Elba Mauriz

**Affiliations:** 1 Department of Nursing and Physiotherapy, Campus de Vegazana, Universidad de León, s/n, 24071 León, Spain; elba.mauriz@unileon.es; Tel.: +34-987-293617; 2 Institute of Food Science and Technology (ICTAL), La Serna 58, 24007 León, Spain

**Keywords:** colorimetric sensor, plasmonics, LSPR, naked eye, nanomaterials, clinical diagnostics

## Abstract

Colorimetric analysis has become of great importance in recent years to improve the operationalization of plasmonic-based biosensors. The unique properties of nanomaterials have enabled the development of a variety of plasmonics applications on the basis of the colorimetric sensing provided by metal nanoparticles. In particular, the extinction of localized surface plasmon resonance (LSPR) in the visible range has permitted the exploitation of LSPR colorimetric-based biosensors as powerful tools for clinical diagnostics and drug monitoring. This review summarizes recent progress in the biochemical monitoring of clinical biomarkers by ultrasensitive plasmonic colorimetric strategies according to the distance- or the morphology/size-dependent sensing modes. The potential of colorimetric nanosensors as point of care devices from the perspective of naked-eye detection is comprehensively discussed for a broad range of analytes including pharmaceuticals, proteins, carbohydrates, nucleic acids, bacteria, and viruses such as Severe Acute Respiratory Syndrome Coronavirus 2 (SARS-CoV-2). The practical suitability of plasmonic-based colorimetric assays for the rapid visual readout in biological samples, considering current challenges and future perspectives, is also reviewed.

## 1. Introduction

The search for rapid disease diagnostic technologies has prompted the development of a great number of in vitro diagnostic methods (over 40,000 products) relying on both laboratory-based analysis and near-patient testing [1]. Although clinical laboratory tests have permitted the detection of a vast majority of analytes, the demand for mobile health applications and non-technician assays has triggered the advancement of healthcare devices at the point of patient care (POC) [2,3,4,5]. Likewise, the effective management of recent disease outbreaks, such as the global pandemic of coronavirus disease 2019 (COVID-19), requires further action to ensure the implementation of efficient diagnostic tools in decentralized settings [6]. From this perspective, point of care testing (POCT) performed outside the laboratory can help to provide timely monitoring of a broad range of conditions without the need for sophisticated instrumentation and skilled operators [7,8,9,10].

Typically, POCT requires handheld devices capable of fulfilling the ASSURED (affordable, sensitive, specific, user-friendly, rapid and robust, equipment-free and deliverable to end users) criteria guidelines of World Health Organization (WHO) for resource-limited environments [1]. POCT has been commonly exploited for molecular diagnostics by electrochemical biosensors (blood glucose meter) and lateral flow assays (LFAs) [11,12]. Nevertheless, the ability to detect analytes of biological interest with sufficient sensitivity and selectivity still remains a challenge for many approaches.

In this framework, colorimetric nanosensors benefitting from the interaction of light and metal nanomaterials have gained importance due to their simplicity and reliability as potential low-cost POC platforms [13,14,15,16]. In comparison to traditional colorimetric assays, nanoparticle-based colorimetric analysis does not require enzymes as biological catalysts and provide color distinguishable changes with the naked eye [17,18]. In particular, the color is generated by the change in absorbance due to the optical properties (metal composition, size, morphology and local environment) of plasmonic nanoparticles [19,20,21,22,23]. As human eyes are unable to detect optical density variations and are only sensitive to spectral peak shifts which produce color variations, these special optical features are of great interest for the quantitative detection of clinical biomarkers by visual inspection [24,25].

**Figure 1:**
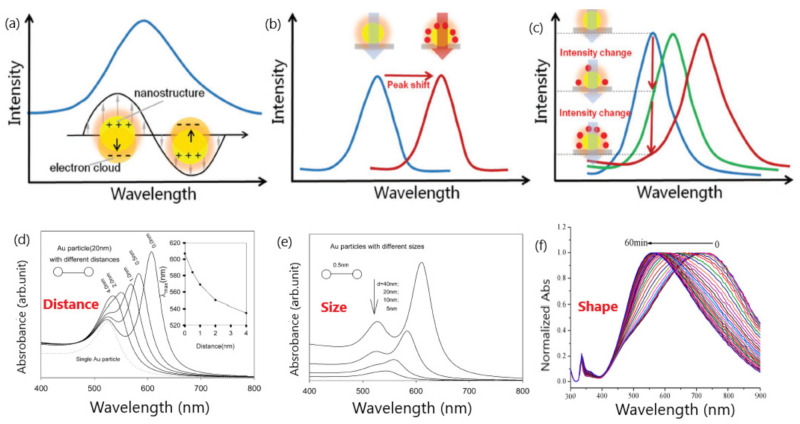
Schematic representation of: (**a**) peak in the extinction spectra of nanostructures as a result of the resonance between the electron clouds and the incident light; (**b**) peak shifts of the spectra due to the changes in refractive index (RI) adjacent to metallic surface induced by the adsorption of target molecules; (**c**) variation of the extinction intensity with the degree of the RI changes near localized surface plasmon resonance (LSPR) sensing substrate at a specific wavelength. Adapted with permission from Wang et al. [3] Copyright © 2017 Wiley; (**d**) change in extinction spectra for 20 nm diameter particles with interparticle distance (s). Inset is the peak shift vs. interparticle distance; (**e**) influence of Au nanoparticle diameter. Adapted with permission from Zhong et al. [22] Copyright © 2004 American Chemical Society; (**f**) SPR (Surface Plasmon Resonance) peak shift of triangular silver nanoprisms in the presence of glucose oxidase (GOD) and glucose. Adapted with permission from Yang et al. [23] Copyright © 2014 American Chemical Society.

Colorimetric sensors take advantage of the localized surface plasmon resonance (LSPR) occurring in the surface of conductive nanoparticles under light stimulation as a result of the confinement of surface plasmons in two-dimensional thin metallic layers [26,27,28]. The LSPR phenomenon enables free electrons to oscillate collectively in the electric field of incident light, leading to the resonance condition [29] (Figure 1). Specifically, gold and silver nanoparticles display LSPR bands within the visible region of the electromagnetic spectrum. This optical property provides the basis for colorimetric sensing, since the intensity, frequency and location of the LSPR bands are highly dependent on the size, shape, surface coating, dielectric environment and aggregation state of metal nanoparticles and therefore can be utilized to detect color changes in colloid suspensions [15,30]. For instance, increments in the size of noble metal nanoparticles generate red-shifts due to the transformation of the LSPR spectral change to a longer wavelength [31]. Likewise, variations in the LSPR bands resulting in red or blue shifts can be observed after nanoparticle aggregation, whilst secondary LSPR bands can be developed at different wavelengths when assembling gold nanoparticles [16].

**Figure 2:**
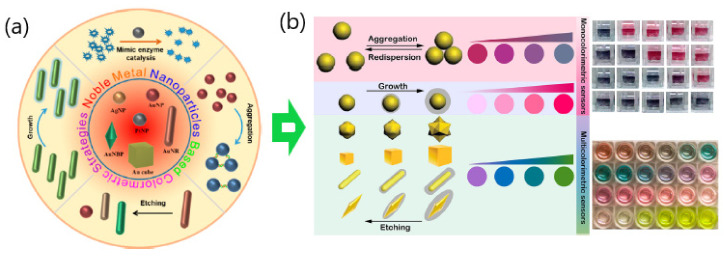
(**a**) Scheme of noble metal nanoparticle based colorimetric strategies Adapted with permission from Ma et al. [32] Copyright © 2019 American Chemical Society; (**b**) monocolorimetric and multicolorimetric signal readouts. Adapted with permission from Wang et al. [16] Copyright © 2019 Elsevier. Adapted with permission from Santopolo et al. [37] Copyright © 2019 American Chemical Society. Adapted with permission from Ma et al. [32] Copyright © 2019 American Chemical Society.

Consequently, the fabrication of colorimetric plasmonic sensors relies mainly on the strong LSPR extinction of metal nanoparticles in the visible range [32,33]. This implies that the extinction coefficients of gold nanoparticles are 1000 times higher than those of traditional organic dye probe-based colorimetric substrates thereby exhibiting sensitivities in the nanomolar range along with more practicality as color readout sensors [34]. Thus, nanomaterials not only offer the possibility of detecting color changes at ultra-low concentrations but also the potential for signal amplification using well suited readout methods [16,35,36]. In general, there are two main colorimetric signal generation mechanisms relying on the interparticle distance or the morphology/size between metal nanoparticles. The distance-dependent color change strategy takes advantage of the cross-linking assembly effect resulting from the aggregation of nearby nanoparticles, whereas the latter one depends on the growth and etching processes caused by the modulation of the dimension and dielectric environment of metal nanoparticles (Figure 2) [14,15,16]. 

Both nanomaterials-based colorimetric approaches have been exploited to monitor chemical or biological species [34], including anions [38], metal ions [39,40], proteins [41], DNA [25], and organic molecules such as glucose [42,43]. Although most naked-eye colorimetric applications are based on the so-called ‘aggregation’ or distance-dependent strategies, plasmonic colorimetric sensors that make use of ‘non-aggregation’ mediated procedures are giving rise to the next generation of clinical diagnostic methods. These interesting features in addition to their capacity for simple production and storage place colorimetric plasmonic nanosensors at the forefront of instruments for site-specific deployment of POCT. 

When comparing the performance of plasmonic sensors according to the variation of the LSPR peak, the main advantage of colorimetric sensors relies on the visual color change generated by colloidal nanoparticles [29]. In contrast with plasmonic sensors based on the change in the local refractive index, the coupling of nanoparticles in the presence of the analyte provides significant benefits for clinical analysis. For instance, the monitoring of analyte concentrations by the simple visualization of the color change offers the possibility of reducing the time of analysis through a rapid detection method with the unaided eye.

Previous works have comprehensively reviewed the most popular functionalization procedures employed in colorimetric biosensing according to the distance dependent LSPR properties and the growth/etching processes of noble metal nanoparticles [14,15,16,31,44,45]. Nevertheless, the evaluation of clinical biomarkers for POCT applications from the unique perspective of naked-eye plasmonic colorimetric sensing has not been sufficiently addressed. Therefore, the aim of this review is to present recent developments (since 2016) in colorimetric detection of clinically relevant compounds and therapeutic drugs using noble metal nanoparticles. This work particularly concentrates on the capacity of the sensing mechanism to generate visual distinguishable color changes using either ‘aggregation’ or ‘non-aggregation’ detection strategies. Latest advances in the development of new colorimetric sensing mechanisms rendering promising diagnostic POC tools for the monitoring of molecular responses by visual inspection are specifically reviewed. 

## 2. Distance–Dependent Plasmonic Naked-Eye Colorimetric Assays

The sensing system relying on the inter particle distance is capable of measuring both LSPR spectral shifts and color variations due to the surface plasmon coupling induced by the aggregation and dispersion of nanoparticles [16,41]. In particular, the aggregation and dispersion of nanoparticles with diameters above 3.5 nm can cause shifts in the plasmon bands along with visible color transitions from red to blue and blue to red, respectively [14,46]. The decrease in the particle distance can be controlled by the presence of the target analyte, thereby setting the basis for colorimetric assays. 

LSPR colorimetric sensors based on the aggregation of nanoparticles may provide high sensitivities and functional designs. Thus, the stability of the assemblies from plasmonic nanoparticles can be modulated by either covalent or non-covalent interactions (i.e., electrostatic interactions, hydrophobic forces, hydrogen bonding and specific biological bonding) and are strongly dependent on external conditions such as temperature, pH and buffer solution [47,48]. However, practical applications for naked-eye detection must face poor resolution, especially for lower target concentrations, because the color readouts by visual inspections only make use of monocolor changes [34]. On the other hand, the auto-aggregation of nanoparticles caused by non-target-mediated factors such as pH, temperature, solvent and molecules charges may lead to false positive/negative results [34]. In spite of these potential limitations, many colorimetric applications have reported ultrasensitive detection levels of biologically relevant targets for molecular diagnostic purposes and drug discovery by merely using the unaided eye [13,49,50,51].

Table 1 presents ‘aggregation’ colorimetric applications that are classified according to the type of clinical biomarker. Several approaches concerning drug monitoring and development are also considered.

### 2.1. Protein-Related and Other Biological Analytes

For instance, clinical diagnostics of physio-pathological processes involving inflammation, tumor growth and metastasis have been performed using biomarkers such as thrombin, a coagulation protein associated with coagulation abnormalities. Lee et al. developed a solid phase colorimetric aptasensor comprising the aggregation of gold nanoparticles (AuNPs) deposited on 3-aminopropyltriethoxysilane (APTES) substrates to visually detect thrombin in 100-fold diluted human serum [52]. The colorimetric sensing exploited a dual amplification method consisting of a sandwich assay with two aptamers (TBA1 and TBA2). First, the TBA1 aptamer was attached to the surface of AuNPs via electrostatic interactions, causing a red to purple color change upon incubation with thrombin. Secondly, the addition of TBA2 conjugated with AuNPs generated a redshift in the absorption peak, owing to the role as a linker of thrombin molecules between the two aptamers (AuNP-TBA1-thrombin-AuNP-TBA2). The interparticle crosslinking is explained because the strong binding between the thiol terminal groups of the aptamers and the AuNPs can weaken the electrostatic interaction between the AuNPs and the substrate. The signal enhancement provided by the formation of a sandwich complex between the two aptamers allows naked-eye detection of thrombin concentrations at 3 μg mL^−1^ with no analytical equipment, whereas a limit of detection of 1.33 μg mL^−1^ was obtained using UV–vis. The reliability of the colorimetric platform was also validated by examining the selectivity in the presence of interfering analytes and the recovery from real biological human serum samples (97.4%).

**Figure 3:**
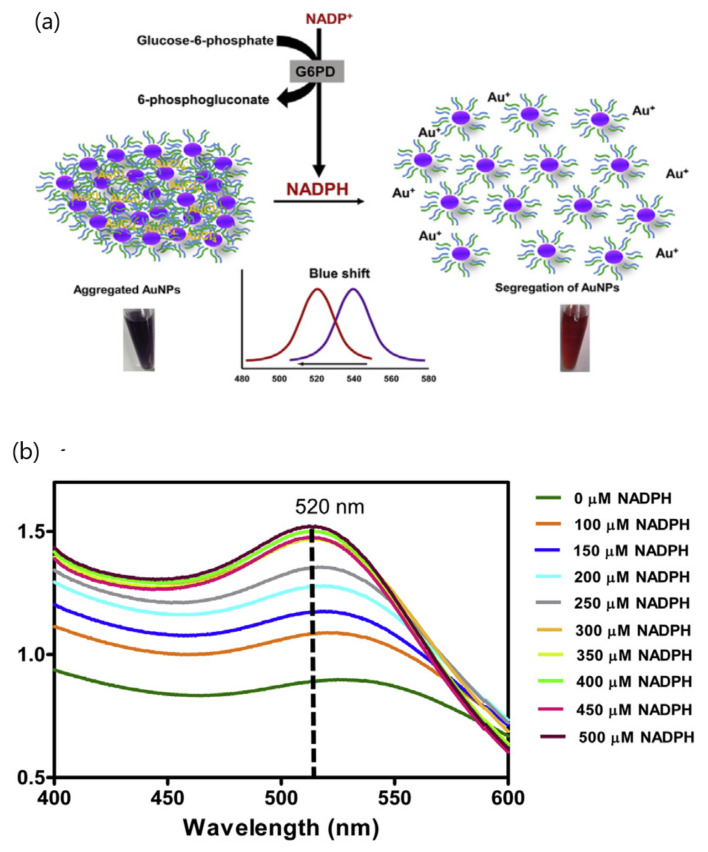
(**a**) Representation of the segregation mechanism of polyvinylpyrrolidone/N,N0-dimethylaminopyridine-stabilized gold nanoparticle by NADPH and (**b**) absorption spectra of gold nanoparticles (AuNPs) in the presence of 0.1 mM HAuCl_4_ and various concentrations of NADPH. Adapted with permission from Boonyuen et al. [53] Copyright © (2020) Elsevier.

A colorimetric assay based on the reversible assembly of gold nanoparticles investigated the differences in the activity of 11 recombinant glucose-6-phosphate dehydrogenase (G6PD) variants [53]. The segregation of AuNPs is mediated by G6PD through the formation of NADPH (Figure 3). In particular, G6PD can control the reduction of NADP^+^ in the presence of complexes of AuCl_4_ and AuNPs (absorbance at 523 nm), yielding a visible color change from bluish to red. The spectral changes of AuNPs varied depending on the enzyme activity of each G6PD variant, thereby generating different amounts of NADPH and allowing the diagnosis of G6PD deficiency by the naked eye. The selectivity of the assay for detecting G6PD specifically in the absence of NADPH was tested using different chemicals and proteins such as G6P (glucose-6-phosphate), NADP^+^, MgCl_2_, Tris-HCl, bovine serum albumin (BSA) and G6PD proteins. Further development of the analytical performance on the reliability of the proposed method will contribute to enabling the diagnostic testing of glucose-6-phosphate dehydrogenase deficiency in real biological samples.

The analysis of the gastric cancer biomarker microRNA-148a has been performed by means of a colorimetric sensor that relies on the conjugation of AuNPs with oligonucleotides used as sensing probes (RNAP) [54]. The dispersion/aggregation status of AuNPs is controlled due to the interaction of the conjugated probes with target DNA using a sandwich hybridization assay in which the complementary sequences are recognized in a “tail-to-tail” or “Head-to-Tail (HT)” alignment. The quantification of microRNA-148a was obtained at ~1.9 nM levels causing a color change from red to purple (SPR absorption peak at 530 nm). The specificity of the assay against another RNA samples did not show either the aggregation of AuNPs or a color change of the solution. The reusability of the biosensor was possible by heating AuNPs above the melting point and utilizing the sharp “melting transition” to disassociate the aggregated nanoparticles. This approach means a promising alternative to detect base sequences in clinical diagnosis.

Another interesting approach was presented for the determination of exosome surface proteins to predict the origin of parent tumors by the naked eye [55]. The assay also utilized an aptasensor platform comprising the formation of complexes between AuNPs and a panel of aptamers that prevented AuNPs aggregation in high salt conditions. Nevertheless, the binding between AuNPs and aptamers could be displaced by the stronger aptamer attachment to the exosome surface proteins that results in the aggregation of AuNPs and the generation of color changes. The red to blue transition of AuNPs enabled the identification of multiple proteins on different cancer cell exosomes, demonstrating their potential as noninvasive diagnostic biomarkers. In addition, by monitoring the interaction between aptamers and AuNPs in the presence of exosomes, visually and quantitively, signature-based exosomal protein patterns were obtained in a single measurement. Further application in clinical specimens could prove the reliability of the proposed method as a POC tool for screening and profiling subtle exosome surface protein differences.

**Figure 4:**
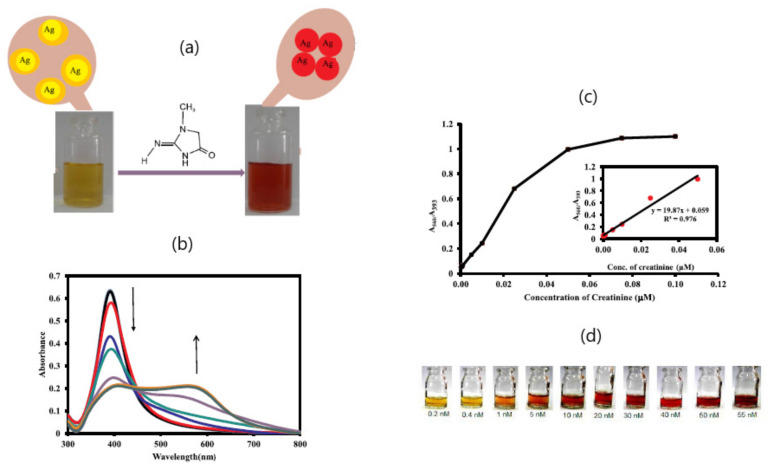
(**a**) Schematic representation of creatinine induced aggregation GA@Ag NPs; (**b**) the UV–Vis. absorption spectra of GA@Ag NPs in the presence of different concentration of creatinine (0.1 to 100 nM); (**c**) corresponding calibration plot of A_560_/A_393_ vs. creatinine concentration (inset shows the linear concentration range); (**d**) photograph of color change of GA@Ag NPs with various concentration of creatinine. Adapted with permission from Sadeghi et al. [56] Copyright © (2020) Elsevier.

The monitoring of creatinine, a primary biomarker of kidney function circulating in blood serum and urine, has been performed by making use of the aggregation of sodium gluconate-capped Ag nanoparticles (GA@AgNPs) [56]. Naked-eye detection was observed after creatinine-induced aggregation of GA@AgNPs, yielding distinguishable color changes from yellow to red (Figure 4). By using an extinction spectra ratio of A_560_/A_393_, creatinine was detected at 0.2 nM with a 0.3–50 nM linear range. The lack of response toward possible interferents was explained by the stability provided through the adsorption of gluconate onto the surface of AgNPs along with the ability to form hydrogen bonds between the target analyte and the hydroxyl groups of gluconates. Additionally, the reliability of the assay was examined by evaluating both the reproducibility and the analytical performance in human and urine plasma samples. The good recovery results demonstrate the potential of the proposed method as a POC tool to quantify creatinine levels in real biological samples by simple visual inspection without requiring sample pretreatment and additional equipment.

**Figure 5:**
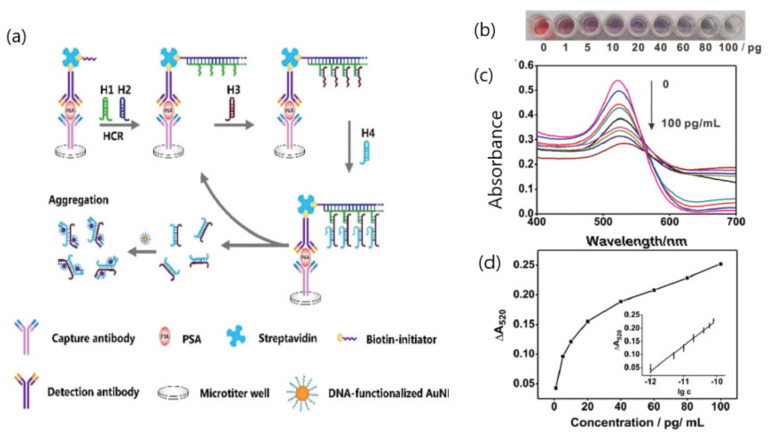
(**a**) Principle of the DNA-programmed plasmonic ELISA for the prostate specific antigen (PSA) detection with the naked eye; (**b**) color changes in the solution; (**c**) corresponding absorption spectrum responses; and (**d**) corresponding ΔA_520_ signals of the developed method for the PSA detection from 1 to 100 pg mL^−1^. Error bars show the standard deviations of three independent measurements. Adapted with permission from Cheng et al. [57] Copyright © (2020) Royal Society of Chemistry.

A plasmonic-based ELISA (Enzyme-Linked ImmunoSorbent Assay) was reported for the detection of the prostate specific antigen (PSA) (model cancer biomarker) using two enzyme-free and isothermal nucleic acid amplification methods: hybridization chain reaction (HCR) and catalyzed hairpin assembly (CHA) [57]. Plasmonic ELISAs provide significant advantages over conventional colorimetric ELISA allowing that the biocatalytic cycle of the enzyme label mediate the LSPR of gold nanoparticles. The assay made use of a biotin-labeled DNA probe that could trigger HCR and CHA processes and induce the aggregation of AuNPs. The hybridization of the reaction products with DNA-modified AuNPs lead to the subsequent color change from red to a color depending on the concentration of the target PSA (Figure 5). The limit of detection (1 pg mL^−1^) was defined as the lowest PSA concentration that could be differentiated by a color change. The quantitative detection was determined by correlating the decrease in the absorbance signal at 520 nm with different PSA concentrations, being three orders of magnitude lower than that of conventional ELISA (1 ng mL^−1^) due to the dual amplification methods. The assay selectivity was examined with regard to 1 ng mL^−1^ carcinoembryonic antigen (CEA) and alpha-fetoprotein (AFP), showing only slight absorbance signal changes at 520 nm. Additionally, the capability for POC diagnosis was evaluated using five clinical serum samples from 1 to 10 pg mL^−1^.

The naked-eye colorimetric detection of cysteine, a supplementary amino acid involved in the regulation of metabolism and utilized as a biomarker of physiological functions and several medical conditions, was described using AuNPs functionalized with β-cyclodextrin (β-CD) [58]. The biosensing strategy exploited the interaction between cysteine and β-CD AuNPs. In particular, the formation of strong Au-thiol bonds via the thiol group of cysteine molecules induced the aggregation of AuNPs, allowing the visualization of color variations from wine red to purple as well as the spectral redshift of the SPR band. The quantification of cysteine was performed in 100-fold diluted human urine and blood serum exhibiting excellent recovery rates (100.22% to 103.35%). The amount of cysteine was calculated with detection limit of 25.47 × 10^−9^ mol dm^−3^, whereas the assay specificity was tested over 19 essential amino acids by the naked eye at 15-fold higher concentration levels, showing high selectivity for cysteine detection. The visual detection of cysteine was complemented with a color scan application provided by a smartphone-based colorimeter detector that proved its potential for the implementation as a remote personalized point-of-care device.

Arginine is another amino acid of special interest in the study of glucose metabolism and diabetes-related diseases, since it stimulates insulin and glucagon secretion [59]. A colorimetric sensor based on four biosynthesized AuNPs from extract of pomegranate plant (*Punica granatum*) that could aggregate in the presence of arginine. The characterization of the assay involved the optimization of the effect of size and shape of AuNPs and pH of the medium. The shift of the SPR was induced by the interaction of the functional groups of the pomegranate attached to the AuNPs with the amino groups of arginine. This interaction lead to the reduction in the distance between AuNPs and their subsequent aggregation causing the visible color change of the solution [violet to brown (AuNP1), red to brown (AuNP2), blue to black (AuNP3) and ash to black (AuNP4)]. The detection limit was in the 10^−6^ M range, showing no detectable changes in the presence of other amino acids. Thus, this work proposed an interesting method for arginine detection using biogenic gold nanoparticles (AuNPs), as a novel alternative to surface modification of AuNPs based on chemical procedures.

A different type of nanoparticle functionalization consisting of glutamic acid (Glu) and polyethylenimine (PE) was utilized for capping the surface of gold nanoparticles (PE-Glu-AuNPs) [60]. The physico-chemical properties of the PE-Glu-AuNPs complex permitted the simultaneous determination of two β-agonists (clenbuterol and ractopamine) by naked eye due to the high stability provided via the dual functionalization approach. The detection of clenbuterol and ractopamine benefited from the interaction with the free amine functional groups of glutamic acid and polyethylenimine to induce rapid aggregation of nanoparticles and a visual detectable color change from wine red to purple blue. The absorbance ratio (A_608_/A_522_ and A_693_/A_522_) changes of PE-Glu-AuNPs were monitored in the presence of different concentrations of clenbuterol and ractopamine displaying detection limits at sub nanomolar levels. On the other hand, the practical application of the colorimetric assay in human urine samples demonstrated that PE-Glu-AuNPs could be used for the detection of clenbuterol and ractopamine by the naked eye for concentrations starting at 200 nM. Since PE-Glu-AuNPs evidenced a better selectivity at pH 5, the specificity of the assay was tested at pH 5 using potentially interferent compounds (alanine, phenylalanine, NaCl, CaCl2, threonine, cysteine, glycine, glucose, salbutamol and urea), showing higher selectivity to clenbuterol and ractopamine.

### 2.2. Infectious Diseases Biomarkers

The screening of infectious diseases is a critical aspect to prevent the spread of pathogens and the outbreak of global emerging pandemics. The application of naked-eye colorimetric detection as early diagnostic methods offers a challenging opportunity to control the transmission of this type of illness and conditions. To date, several studies have reported analytical methods for monitoring bacteria and virus using colorimetric nanosensors based on the aggregation/dispersion of nanoparticles.

**Figure 6:**
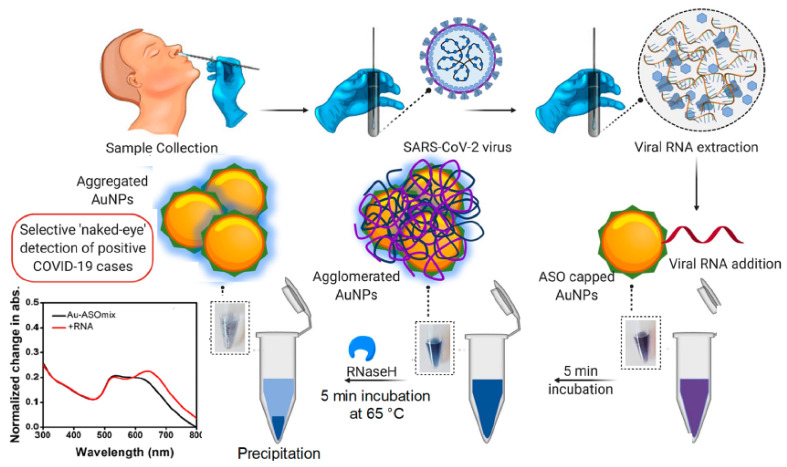
Schematic representation for the selective ‘naked-eye’ detection of SARS-CoV-2 RNA mediated by the suitably designed antisense oligonucleotides (ASOs), capped AuNPs and normalized change in absorbance of the gold nanoparticles before and after the addition of total RNA containing the SARS-CoV-2 viral load. Adapted with permission from Moitra et al. [61] Copyright © (2020) American Chemical Society.

A novel colorimetric assay has been developed by Moitra et al. for the detection of SARS-CoV-2, an enveloped non-segmented β-coronavirus, that causes the new severe acute respiratory syndrome and coronavirus disease (COVID-19) [61]. The method took advantage of the positive-sense, single-stranded RNA of SARS-CoV-2 to functionalize AuNPs with thiol-modified antisense oligonucleotides (ASOs), specific for N-gene (nucleocapsid phosphoprotein). The aggregation of AuNPs was observed in the presence of its target RNA sequence generating a red shift of ~40 nm in the SPR absorbance spectra (660 nm wavelength) and visual color variations from violet to dark blue (Figure 6). Additionally, the cleaving of the RNA strand from the composite hybrid of RNA and Au-ASO composite yielded a naked eye-detectable color change in the presence of endonuclease RNAse H. A limit of detection of 0.18 ng μL^−1^ of RNA was obtained for SARS-CoV-2 viral load in the presence of MERS-CoV viral RNA, displaying the selectivity of the method for detecting its target. The assay benefits from the capacity for targeting the N-gene regions of SARS-CoV-2 simultaneously at multiple positions, thereby enabling the possibility of monitoring viral gene mutations during its current spread. Therefore, it provides a feasible alternative for the early diagnosis of COVID-19 in comparison with the commercial diagnostic methods approved or under revision by the FDA (Food and Drug Administration) through simply detecting the presence of SARS-CoV-2 by the naked eye.

**Figure 7:**
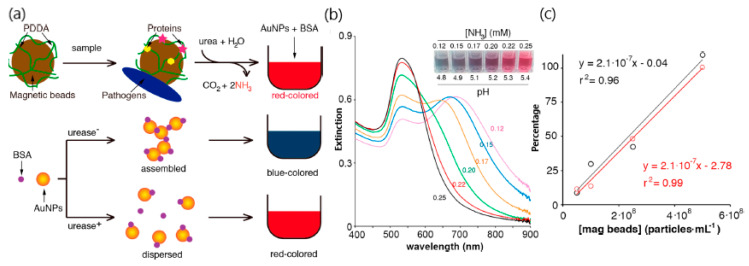
(**a**) Schematic representation of the method used for detecting urease-positive bacteria: Proteins and pathogens are physically adsorbed on magnetic microparticles covered with poly-(diallyl dimethylammonium chloride (PDDA); the urease molecules and urease-producing bacteria captured this way are then specifically detected by adding urea, which generates red colored tests. The signal generation step consists of adding bovine serum albumin (BSA) and gold nanoparticles (AuNPs) to the urea solution; in the absence of urease, BSA triggers the formation of nanoparticle aggregates with red-shifted LSPR; in the presence of urease, NH_3_ is generated, which prevents nanoparticle aggregation; (**b**) extinction spectra after mixing 500 μL of AuNPs with 500 μL of 1 μg mL^−1^ BSA supplemented with (**a**) NH3 and (**c**) percentage urease activity recovered with to PDDA-coated magnetic beads after adding the particles to a solution containing urease in phosphate buffer with a concentration of 0.1 (black) or 0.01 (red) μg mL^−1^. Adapted with permission form Santopolo et al. [37] Copyright © (2019) American Chemical Society.

In addition to the risk of virus outbreaks, the spread of antimicrobial resistances poses a significant threat for public health that should be controlled by the identification of bacterial strains in a short period of time. Therefore, visual detection of the activity of bacterial enzymes seems to be a promising approach for personalized bacterial monitoring at the point of care. In this framework, a plasmonic colorimetric approach demonstrated the detection of urease-producing bacteria via a capture procedure relying on the interaction of positively charged polymer poly-(diallyl dimethylammonium chloride) (PDDA) magnetic beads with negatively charged bacteria or proteins [37]. The addition of bovine serum albumin (BSA) and gold nanoparticles (AuNPs) to the solution containing PDDA-covered magnetic beads and urea generates the color change (Figure 7). The presence of urease positive bacteria can increase the pH and hydrolyze urea by the formation of NH_3_, thus preventing nanoparticle aggregation and inducing red color variations. Conversely, urease-negative bacteria do not increase the pH of the urea solution, being acidic enough for BSA to enable the assembly of gold nanoparticles and the changing of the color solution to blue. Therefore, a distinctive color evolution from red−mauve to gray−blue−violet colors can be triggered by the assembly of gold nanoparticles in the presence of BSA bovine serum albumin (BSA). Likewise, by changing the pH of the solution through the concentration of NH_3_, the urease positive bacteria *Proteus mirabilis* could be detected in urine with a limit of detection of 10^1^ cells mL^−1^ even in the presence of the urease negative bacteria Pseudomonas aeruginosa. These features increase their capacity as point of care devices.

A similar approach consisting of the utilization of magnetic microbeads to capture AuNPs clusters has been reported for the detection of DNA colorimetrically [62]. The sensing mechanism allowed the amplification of the plasmonic signal by the cascade event triggered by the DNA target molecule. A pink/red color change was induced by the presence of the target due to the capturing of AuNPs, reaching high sensitivity at attomole levels by naked eye. The method was applied to target a specific region of the E. coli genome enabling lower detection levels (7.5 × 10^2^ CFU/μL) by visual inspection in comparison with conventional PCR methods (3.8 × 10^3^ CFU/μL), demonstrating its ability for POCT.

The enzyme-mediated growth of AuNPs was utilized for the analysis of respiratory syncytial virus by using a dual-signal amplified detection method [63]. This work exploits the dispersion of aggregated AuNPs triggered by alkaline phosphatase combined with the loading capacity of magnetic beads and the stimulation effect of zinc ion for signal enhancement. Specifically, magnetic beads were capable of carrying thousands of enzyme molecules while the dephosphorylation reaction is accelerated owing to the introduction of Zn^2+^ metal ions. The utilization of enzymes to disaggregate AuNPs reduced the risk of false positive signals allowing the naked-eye detection by visualizing a color change color upon variation of the RSV concentration. In particular, a limit of detection of 0.021 and 0.035 pg mL^−1^ were found for RSV in PBS buffer and spiked serum samples, displaying a distinguishable color variation from gray to red with the increase in RSV concentration. Therefore, this reverse colorimetric assay proved to be 50 times more sensitive than conventional ELISA, seeming an interesting alternative for pathogen detection.

### 2.3. Drug-Induced Aggregation

Therapeutic drug monitoring often requires timely measuring of specific drugs at different intervals to ensure constant blood concentrations whilst surveilling patient adherence to treatment [50]. In this context, colorimetric assays offer unique opportunities for the remote biosensing of clinical effects and systemic toxicity in order to optimize drug dosage and patient response at the point of care [51]. The detection of antidepressants is of special interest to manage the relationship between pharmaceutical preparation and therapeutic effects in biological fluids. In this sense, the monitoring of fluoxetine was reported with a colorimetric sensor using citrate-capped silver nanoparticles (CIT-Ag NPs) [64]. The presence of fluoxetine in the solution induced the aggregation of CIT-Ag NPs, causing a color change from yellow to dark brown with an absorption peak at 400 nm and red shifted of the surface plasmon band. The effects of several conditions (pH, time) along with analytical parameters such as intra and inter-day variability and selectivity over pharmaceutical additives were also studied. Under optimized conditions, a limit of detection of 0.18 μg mL^−1^ and a limit of quantification of 0.54 μg mL^−1^ were obtained for a concentration range of 2–10 μg mL^−1^. The potential of the assay for clinical analysis was demonstrated by showing the lack of interference of biological fluids in spiked human urine and blood serum samples, rendering 97.75 to 98.5% recoveries.

Another application that made use of silver nanoparticles (AgNP) as visual probes has been developed to detect metformin, an anti-diabetic drug with anticancer and antiaging effects [65]. The modification of AgNOs with cucurbit(6)uril CB(6) enabled the recognition of metformin at micromolar levels. The presence of metformin led to the aggregation of CB(6)-modified AgNPs and subsequently to changes in the color of the solution and the absorption spectrum of nanoparticles. The utilization of CB(6) as supramolecular host molecule allowed the attainment of metformin detection levels in the 3 to 750 μM range with a limit of detection of 1 and 75 μM using spectrophotometric (absorbance ratios A_550_/A_400_) and visual identification, respectively. The selectivity and reliability of the method was also determined adding (Na^+^, K^+^, Cl^−^, SO^2−^, CO_3_^2−^) and some substances (urea, glucose) normally present in urine samples, displaying both good selectivity and sensitivity values. Only a slight increase in the absorbance ratio was observed for urea, whereas good recoveries (95–115%) were obtained in 50 times diluted urine samples. Thus, the introduction of the molecular recognition mechanisms seems to provide a promising method for metformin detection.

The exploitation of unmodified silver nanoparticles has also been applied to the detection of a non-chromophoric drug-azithromycin, a macrolide antibiotic with high activity against Gram-negative and Gram-positive organisms [66]. The utilization of citrate-capped AgNPs helped to stabilize the assay, since positively charged azithromycin contributed to counteracting the negative charge on the particle surface thus inducing particle aggregation and a distinct color change from yellow to red-brown and then to purple for azithromycin concentrations ranging from 0.2 to 100 μM, The analysis of the factors affecting the assay response such as pH, volume of AgNPs suspension and incubation along with the effect of several anions, cations and other antibiotics were characterized to optimize the assay conditions. The linear range was between 0.2 and 100.0 μM, while the reliability of the colorimetric assay in pharmaceutical formulations and human plasma displayed good precision and recovery rates (98.74–101.34%).

## 3. Colorimetric Sensors Based on Etching and Growth of Noble Metal Nanoparticles

The term ‘non aggregation’ is usually applied to colorimetric sensors that depend on the morphology and size of noble metal nanoparticles for detecting target analytes. In comparison to classical nanoparticle-based aggregation methods, ‘non aggregation’ colorimetric sensors are reported to improve the sensing readout by making use of specific catalytic conversion processes (chemical or biological) based on monocolor or multicolor variations. Another important merit is the possibility of avoiding false/positive results due to the stability of the color substrate and the ability to prevent both auto-aggregation and the interference of foulants in complex biological matrices [16]. The possibility of developing colorimetric sensors based on test strips are further advantages over other plasmonic sensing schemes [14,15,44]. These features become essential for clinical analysis, thereby setting the fabrication of ‘non aggregation’ platforms in a prominent position for the development of POCT applications.

**Figure 8:**
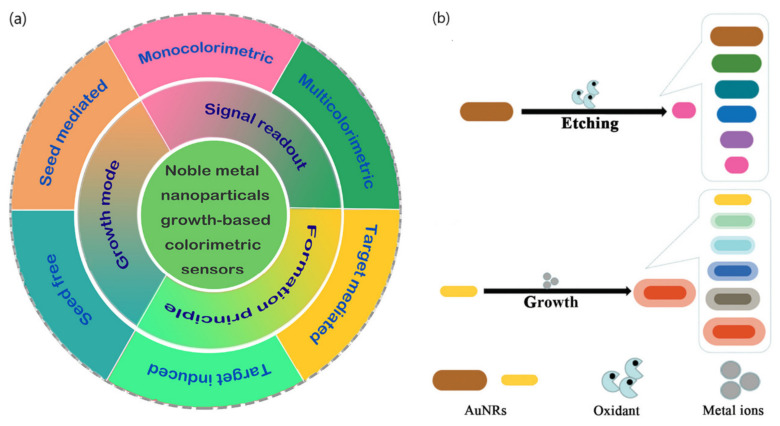
(**a**) Schematic representation of seed-free and seed-mediated based colorimetric sensors which depend on different signal generation mechanisms. Target-induced and target-mediated colorimetric protocols which rely on different sensing principles. Monocolorimetric and multicolorimetric sensors based on different signal readouts. Adapted with permission from Wang et al. [16] Copyright © 2019 Elsevier; (**b**) two routes for the evolution of the shape of AuNRs used in AuNR multicolorimetric assays: AuNR-etching based process and AuNRs growth-based process. Adapted with permission from Rao et al. [44] Copyright © 2019 The Royal Society of Chemistry.

According to the sensing mechanism, ‘non aggregation’ colorimetric sensors can be classified in relation to the growth and etching of metal nanoparticles. On the one hand, the nanoparticles’ growth implies the production of new nanostructures that yield changes in the extinction spectra of the LSPR band and the color of the solution [14,16]. The growth of newly generated nanoparticles is induced by metal ions in the absence or presence of seeds and can be determined by different signal readouts (monocolorimetric, multicolorimetric), generation mechanisms (seed-free, seed-mediated) and sensing principles (target-induced/mediated) (Figure 8) [16]. The utilization of different precursors, reducing or capping agents for the generation of nanoparticles can also contribute to modifying LSPR properties, yielding different visual color readouts.

In contrast, etching processes are associated with the change in color and the shift of the LSPR band extinction spectra resulting from the tuning of the size, shape, and composition of nanoparticles. By controlling the morphology/size and kinetics of nanoparticles in the presence of the etchant, usually through oxidation, the recognition events induced by the target can generate a variation of LSPR and detected by naked-eye [15]. Additionally, etching-based colorimetric sensors can benefit from enzyme-mediated strategies in which the products of a reaction catalyzed by an enzyme can induce the etching of metal nanoparticles, thus offering further applications in plasmonic DNA assays and enzyme linked immunosorbent assays (ELISAs).

Therefore, this section presents recent development on growth/etching colorimetric biosensing strategies on the basis of the type of clinical biomarker employed in the application (Table 2).

### 3.1. Colorimetric Glucose Detection

Glucose is the primary biomarker of diabetes mellitus, the most common metabolic disorder. The monitoring of glucose levels at designed time intervals is required to prevent abnormal concentrations in biological fluids. Therefore, the accurate and continuous testing of glucose levels in blood and urine is mandatory to keep diabetes under control and avoid serious clinical complications. Although the most popular glucose biosensor utilizes an electrochemical sensing scheme based on screen-printed electrodes, several colorimetric nanosensors have proved to be valuable prognostic methods for monitoring glucose in either blood or urine samples by naked-eye.

**Figure 9:**
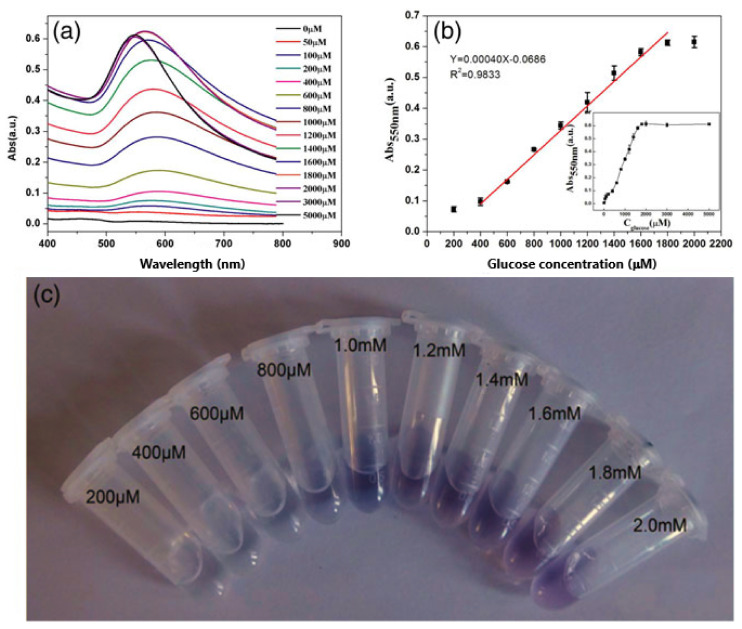
The UV–vis absorption spectra (**a**), the linear fitting of A_550_ and glucose (**b**) and the images (**c**) with varied concentrations of glucose. Inset of (**a**) shows the relation of A_550_ and different concentrations of glucose. Error bars show the standard deviations of measurements taken from three independent experiments. Adapted with permission from Weng et al. [67] Copyright © 2017 Wiley.

The majority of colorimetric assays that measure glucose using metal nanoparticles rely on etching-based approaches. Nevertheless, a biosensing design making use of in-situ growth of Au NPs allowed the sensitive detection of glucose with a detection limit of 6.28 μM (AuNPs absorbance 550 nm) [67]. First, glucose was oxidized by the enzyme glucose oxidase whereas the product of the reaction, H_2_O_2_, was used as the detecting target molecule. The stabilization of the assay was achieved using Tween 20, exhibiting distinct colors due to the reduction of HAuCl_4_ by H_2_O_2_. Under optimized conditions, the presence of glucose in the solution caused a visual detectable change from colorless to red. A blue shift was observed when increasing glucose concentrations due to the formation of gold particles with non-aggregated pattern and red appearance (Figure 9). Although the proposed method did not prove its applicability in clinical specimens, good selectivity values where obtained by measuring the interference of various compounds normally found in beverages such as sugars, food additives, and amino acids.

Clinical diagnosis of glucose molecules in 1:1000 diluted samples was performed using a biocatalytic method based on the shape-altering of gold nanostars (AuNSs) generated by the deposition of silver [68]. In particular, the silver ions coated onto the AuNSs produced the shape alteration as a result of the oxidation of glucose. The change in morphology led to the formation of nanospheres and different color variations depending on the concentration of glucose and the sample matrix (2-(N-morpholino) ethane sulfonic acid (MES) buffer or serum). The evolution of color was clearly differentiated in both matrices as the concentration of glucose increased. Thus, the color varied from blue to purple to orange from the control to the highest glucose concentration in MES buffer (131 nm shift), whereas in serum the color changed from blue to intensified blue to deep purple (80 nm blueshift). The extent of color change was in accordance with the changes in morphology since the AuNSs changes in MES solution were higher than in serum. The analytical performance was examined by testing the assay specificity with other saccharides, showing a lack of LSPR peak shifts. Therefore, this signal generation mechanism proved its potentiality for the naked-eye detection of glucose in serum samples.

**Figure 10:**
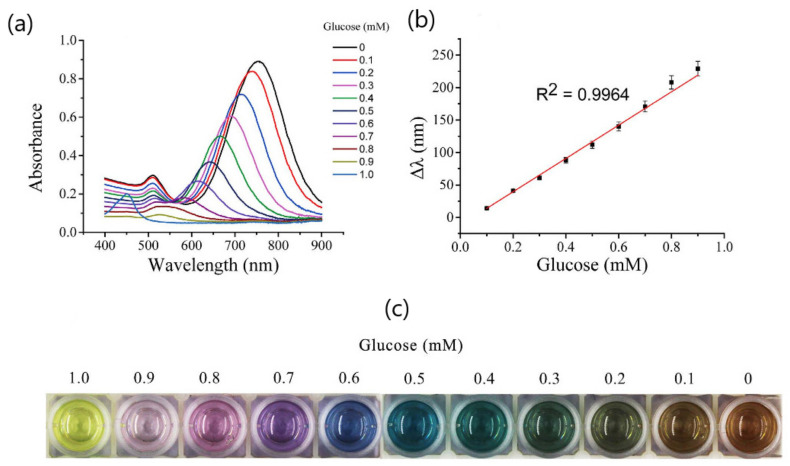
(**a**) The UV-Vis spectra change of AuNRs in the oxidation etching process with different concentrations of glucose; (**b**) the LSPR shift of AuNRs as a function of glucose concentration; (**c**) Color change of the plasmonic sensor with the decrease in glucose concentrations. Adapted with permission from Lin et al. [69] Copyright © 2016 Scientific Reports.

The determination of glucose based on the etching of gold nanoparticles has been performed using different color readouts, signal generation mechanism and sensing principles. For instance, Lin et al. exploited a HRP-H_2_O_2_-3,3′,5,5′-tetramethylbenzidine (TMB) system coupled with an enzymatic reaction to produce H_2_O_2_ in order to etch gold nanorods (AuNRs) [69]. In particular, the product of H_2_O_2_-HRP-TMB-HCl catalyzed oxidation system, 3,3′,5,5′-tetramethylbenzidine(II) (TMB^2+^) was used to detect glucose by measuring vivid color changes. The visual sensor comprised a multicolorimetric signal readout that utilized TMB^2+^-AuNRs as chromogenic substrate for the quantitative detection of glucose by the naked eye. The signal generation resulted from the completely oxidation of AuNRs enables that color changes to yellow due to only TMB^2+^ exists. The correlation between the gradual longitudinal blue shift of LSPR and glucose concentrations in the 0.1 to 0.9 mM range showed distinguishable vivid colors (from the reddish brown, gray, green, blue, purple, pink to yellow) according to the concentration of glucose in 10-times diluted human serum (Figure 10). The specificity of the assay was examined against a wide range of compounds normally present in serum samples, including fructose, sucrose, ascorbic acid (AA), uric acid (UA) and a series of amino acids (glutamic acid, glycine, histidine, alanine, tyrosine, serine, and phenylalanine). Therefore, the etching of AuNRs by TMB^2+^ proved to be an interesting approach to detecting glucose in human serum by the naked eye.

A similar approach relying on enzymatic-mediated etching of gold nanorods (GNRs), was developed by Zhang et al. for the detection of glucose in urine samples [70]. The assay also used a multicolor signal readout and took advantage of the catalysis of MoO_4_^2−^ to achieve the etching of AuNRs by the H_2_O_2_ generated through the glucose–glucose oxidase enzymatic reaction. A visual detection limit of 3 μM was obtained in buffer solution (0.45 mM urine). To study the applicability in biological samples, 500-fold diluted human urine was spiked with glucose at different concentrations. The color response of AuNRs involved a multicolor change from blue to red to colorless due to the peak blue-shift of longitudinal LSPR bands. Despite achieving excellent sensitivities, the assay did not report other relevant results regarding the analytical performance, such as selectivity and stability. Nonetheless, the comparison of real urine samples of diabetes volunteers with urine samples from healthy volunteers could be easily distinguished.

**Figure 11:**
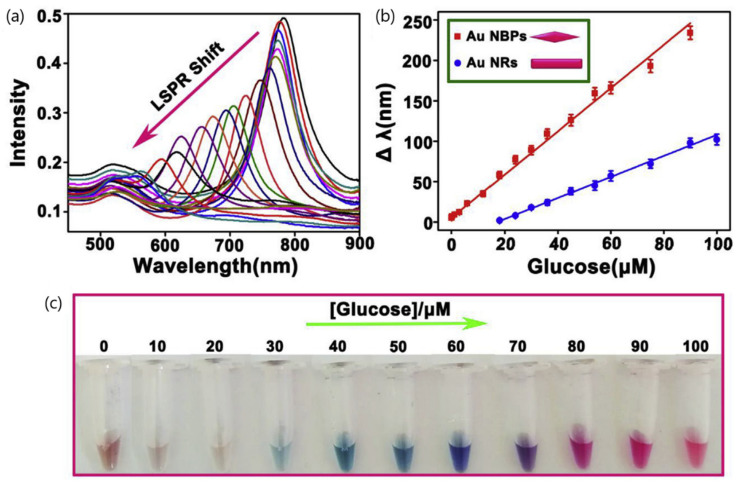
(**a**) UV-vis spectra change of Au NBPs (gold nanobipyramids) in the oxidation etching process in the presence of various concentrations of glucose (from right to left: 0, 0.05, 0.15, 0.3, 1.2, 3, 6, 10, 20, 30, 40, 50, 60, 70, 80, 90, 100, 120, 150, 180 and 200 mM); (**b**) linear curve for glucose detection on the basis of Au NRs (blue line) and Au NBPs (red line), respectively; (**c**) color change of the colorimetric assay with the increasing glucose concentrations. Adapted with permission from Xu et al. [71] Copyright © 2019 Elsevier.

Likewise, another multicolorimetric assay was developed using enzymatic-mediated etching of gold nanobipyramids (AuNBPs) for detecting glucose in blood samples [71]. The sensing principle relies on the catalytic oxidation of H_2_O_2_ (generated from glucose oxidation) by horseradish peroxidase (HRP) to generate hydroxyl radicals (OH). The strong oxidizability of OH groups can accelerate the Au NBPs’ etching and generate vivid colors visually detected and the blue shift of the LSPR band. In comparison to gold nanorods (10 μM), the assay reported better sensitivity (0.02 μM) being three orders of magnitude lower for AuNBPs. The method also exhibited good selectivity with regard to other commonly existing substances in human serum, including uric acid (UA), ascorbic acid (AA), sucrose, fructose, lactose and some common amino acids (histidine, tyrosine, serine, glutamic acid, alanine, phenylalanine and glycine). The ability to detect visual changes by the naked eye was proved using serum samples from healthy people and diabetes patients. Blood glucose levels in the normal range (3.0–6.0 mM) generated green and blue colors and were easily distinguishable from a blood glucose level beyond the normal value (approximately 7.0 mM), which rendered purple or pink colors (Figure 11).

Finally, a singular approach consisting of Au@Ag core–shell nanoparticles (Au@Ag NPs) was applied to the simultaneous detect simultaneously glucose and cholesterol in urine and human serum [72]. The fabrication of (Au@Ag NPs) involved in situ growth of silver nanoparticles (AgNPs) on the surface of thiol-PEG-capped gold nanoparticles (AuNPs, 13 nm). An absorbance peak at 375 nm was observed because of the SPR absorption of Ag NPs. The presence of glucose and cholesterol induced the etching of Au@Ag NPs due to the production of H_2_O_2_ generated from glucose and cholesterol oxidation. The naked-eye detection of glucose and cholesterol was possible owing to the color change from orange to red originated by the decrease in the characteristic absorbance at 375 nm. Under optimized conditions, for glucose and cholesterol were detected at 0.24 and 0.15 mM, respectively. Further application in real human serum and urine samples yielded good recovery results. This feature, along with the demonstrated selectivity tested by fructose, sucrose, lactose, galactose, glycine, methionine, arginine, K^+^, and Na^+^, make this method an interesting and simple alternative to enzymatic-mediated approaches.

### 3.2. Other Biological Analytes

The detection of the prostate specific antigen (PSA) via high-throughput methods has attracted growing interest in recent years, since it is the most accepted biomarker for the early diagnosis of prostate cancer. Accordingly, the monitoring of PSA levels in biological fluids has been addressed using different biosensing strategies including signal generation mechanisms based on colorimetric principles.

**Figure 12:**
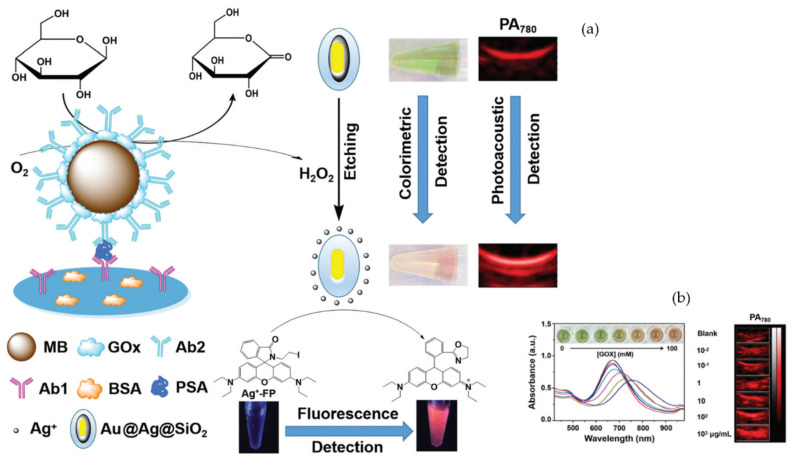
(**a**) Illustration of an Au@Ag@SiO_2_ based dual-round signal amplification ELISA system for colorimetric, photoacoustic (PA) and fluorescence detection of PSA; (**b**) UV-vis absorption spectra, color changes and PA_780_ images. Adapted with permission from Jiang et al. [73] Copyright © 2020 The Royal Society of Chemistry.

In this sense, a singular approach consisting of a silica-coated Au@Ag core–shell nanorod (Au@Ag@SiO_2_)-based ELISA system with triple read-out including colorimetric, fluorescence and photoacoustic detection was used to detect PSA by a dual-round signal amplification strategy [73]. The assay benefitted from the catalytic reaction occurring upon the ELISA format to achieve the effective etching of the Au@Ag@SiO_2_ complex and the production of color changes (Figure 12). Specifically, the PSA in solution is captured by the monoclonal primary antihuman PSA antibody (Ab1) coated on the substrate and then recognized by the secondary antihuman PSA detection antibody (Ab2) conjugated with glucose oxidase (GOx) and functionalized with magnetic beads (MBs), thus providing signal amplification due to their large specific surface area. The conjugated GOx catalyzed the addition of glucose to generate hydrogen peroxide (H_2_O_2_), which etch the Au@Ag@SiO_2_. Therefore, the second amplification was produced by the silver ions (Ag^+^) generated after the etching and degradation of the silver layer within plasmonic Au@Ag@SiO_2_. This degradation can induce a green to pink color change visually detected by the naked eye and a red shift due to SPR absorption, thereby enhancing the photoacoustic read-out signal at 780 nm. The Ag^+^ generated can also enhance the florescence readout as a result of the capture by an Ag^+^ fluorescent probe (Ag^+^-FP). By optimizing the assay conditions, a limit of detection of 0.1 ng mL^−1^ for fluorescence imaging and 1.5 ng mL^−1^ for ELISA. Although the assay specificity or the reliability in biological samples was not reported, the proposed method provides interesting findings regarding three distinct biosensing strategies.

**Figure 13:**
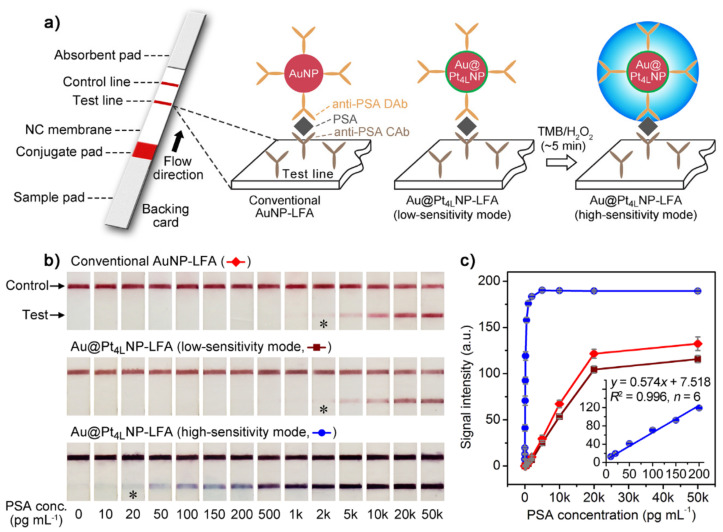
Detection of PSA with a conventional AuNP-based lateral flow assay (AuNP-LFA) and the Au@Pt4L NP-based LFA (Au@Pt_4L_ NP-LFA). (**a**) Schematics showing the principles of AuNP-LFA and Au@Pt_4L_ NP-LFA at low- and high-sensitivity modes (see Supporting Information for details about the preparation of LFAs); (**b**) representative photographs taken from the LFAs of PSA standards. The asterisks (*) indicate detection limits by the naked eyes; (**c**) Corresponding calibration curves of the detection results shown in (**b**). Error bars indicate the standard deviations of six independent measurements. Inset shows the linear range region of the Au@Pt4L NP-LFA at high-sensitivity mode. Adapted with permission from Gao et al. [74] Copyright © 2017 American Chemical Society.

Another strategy that utilizes core–shell AuNPs with dual functions involving both plasmonic and catalytic activities is presented by Gao et al. [74]. This dual functionality was achieved by coating AuNPs with ultrathin Platinum (Au@Pt NPs) skins of sub-10 atomic layers. The catalytic activity of the Au@Pt NPs (high-sensitivity mode) allowed the sensitivity enhancement by generating detectable color signals several orders of magnitude stronger than that provided from plasmonics (low-sensitivity mode), because the intensity of blue color from catalysis was significantly higher than that of the intrinsic red color from plasmonics. The assay was applied as a lateral flow assay (LFA) to detect PSA by comparing the performance of Au@Pt_4L_ NPs and AuNPs LFAs (Figure 13). At the test line, the naked-eye detection limit for PSA was approximately 2 ng mL^−1^ for both AuNP- and Au@Pt_4L_ NP-LFAs (“low-intensity mode”), the intensities of red bands being almost the same. In contrast, the “high-intensity mode” permitted that PSA concentrations of 20 pg mL^−1^ produce resolved blue/purple lines. In addition, the applicability to clinical analysis was demonstrated by measuring PSA from real human plasma samples, yielding good correlation coefficients with regard to conventional ELISA. Likewise, the utilization of test strips could contribute to increasing the stability of nanoparticles while preventing their aggregation.

**Figure 14:**
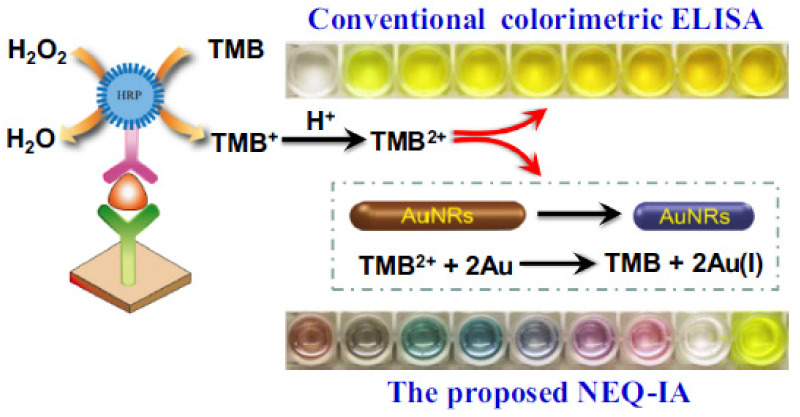
Comparison of the conventional colorimetric ELISA and NEQ-IA ELISA. Adapted with permission from Ma et al. [75] Copyright © 2017 Elsevier.

The combined detection of CEA and PSA was presented by Ma et al. [75] using another etching-based process that comprised the utilization of the oxidized form of 3,3′,5,5′-tetramethylbenzidine (TMB), TMB^2+^, to etch gold nanorods (AuNRs) in the presence of the target analytes. In particular, the assay exploited an immunoassay format that making use of horseradish peroxidase (HRP) as the enzyme label and TMB-AuNRs mixture as the chromogenic substrate allowed the quantitative detection of CEA and PSA by displaying vivid colors in response to concentration variations. Unlike conventional ELISA, wherein the acidic solution is added to display a yellow color, in the present method, the addition of AuNRs generated a series of colorful solutions. The method of quantification implied the construction of a standard reference card to compare the color of the sample to the color of one of the standard color solutions. By using this method, a limit of detection of 2.5 ng mL^−1^ and 75 pg mL^−1^ were obtained for CEA and PSA, respectively (Figure 14). The visual quantification of the target analytes in serum samples was compared with commercial ELISA kits, and the chemiluminescence immunoassay (CLIA) showing good consistency. Although the assay did not include further validation of other analytical parameters, the method was presented as a proof of concept for the visual quantification of clinical biomarkers by simply comparing the solution color with a standard reference card (a colored paper), similarly to pH tests.

The etching of gold nanorods has been also exploited by a multidimensional colorimetric sensor that made use of the oxidization of TMB (colorless) into TMB^2+^ (yellow) through H_2_O_2_ to generate the blue shift of the LSPR band and distinguishable colors (gray, purple, blue and pink), under different TMB^2+^ concentrations [76]. The catalytic activity is affected by the presence of proteins, leading to three distinct sensing elements: the aggregation behavior (A_620_/_520_), the catalytic activity (A_450_) and the peak blueshifts (Δλ) that can be adjusted to allow the discrimination of native and thermally denatured proteins. The assay was applied to the study of conformational changes of fourteen proteins (seven native proteins and seven temperature-induced denatured proteins). The efficiency of the sensor array was demonstrated by the identification of pure and binary mixtures of proteins and subsequent discrimination through hierarchical cluster analysis. In spite of the inherent benefits of the proposed method, the naked-eye detection was only shown for the study of the behavior of AuNPs in the presence of human serum albumin (HSA) and Lys proteins.

Another colorimetric nanosensor based on the etching of gold nanorods exploited the peroxidase-like activity of ethylene diamine tetra acetic acid (EDTA) for the visual read-out of circulating tumor cells [77]. This work takes advantage of the photocatalytic property of EDTA, which was found to be comparable to that of natural horseradish peroxidase (HRP). Under light emission, the decomposition of hydrogen peroxide can be catalyzed by EDTA while regulating the longitudinal plasmon wavelength of AuNRs and producing various color solutions. The assay utilized a sandwich format in which the target molecule interacts with the capture antibodies coated to the substrate and recognized by secondary antibodies labeled with EDTA. The addition of AuNRs and H_2_O_2_ into the sandwich structure generates a color change due to decomposition of H_2_O_2_ by EDTA. In addition, H_2_O_2_ reacts with AuNRs, inducing the modulation of their aspect ratio along with different color variations depending on the concentration of target analyte. The assay enabled the detection of the biomarkers of breast (CA15-3) and prostate (PSA) cancer with high sensitivity (7.52 × 10^−15^ U/mL) and accuracy in human serum samples. The plasmonic peroxidase-like EDTA visual readout was also applied to the monitoring of MCF-7 cancer cell lines by combining immunomagnetic separation of circulating tumor cells (CTC) from blood with anti-EpCAM–coated magnetic beads and visual read-out of CTCs by naked-eye, thus providing a promising method for the diagnosis of cancer-related biomarkers.

**Figure 15:**
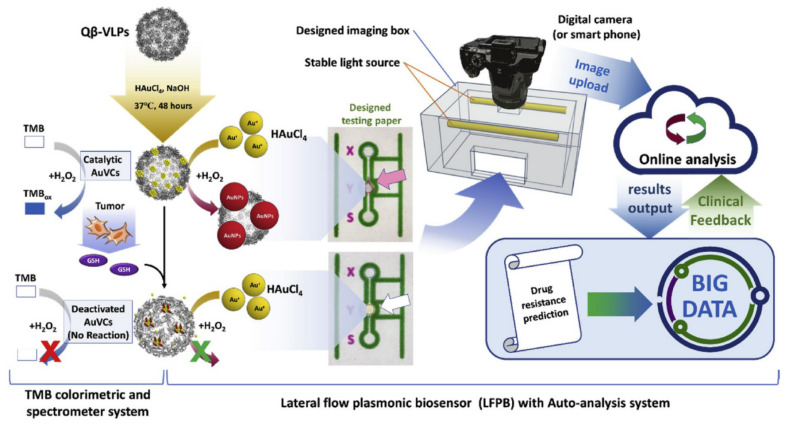
The overview design and procedures of this study. Qβ-VLPs (Virus like particles) were co-incubated with HAuCl_4_ and NaOH to generate AuNCs. The formed AuVCs are glutathione (GSH)-sensitive and could catalyze the formation of AuNPs in the presence of HAuCl_4_ and H_2_O_2_. The deactivation of AuVCs caused the catalysis ability to decrease, leading to the poor yield of AuNP formation. Designed testing paper containing recognition lines and flow-detection zones were printed using a wax printer. The sample could be analyzed using an image box with a smartphone or digital camera then sent data to the online auto-analyzing software. The output data could help in the prediction of drug resistance and for big data analysis to improve the prediction accuracy. Adapted with permission from Pang et al. [78] Copyright © 2020 Elsevier.

Finally, a singular approach allowed the detection of glutathione (GSH) with a lateral flow plasmonic biosensor (LFPB) based on gold-viral biomineralized nanoclusters (AuVCs) used as nanozymes [78]. The sensing mechanism is based on the growth of AuNPs induced by the presence of GSH. The concentration of GSH in solution generates different color patterns that are first visualized with the naked eye and then quantified by a smartphone with auto-analysis software (Figure 15). The limit of detection calculated for GSH was 9.80 μM, whereas the temozolomide (TMZ) drug-resistant levels of glioblastoma multiforme (GBM) cells were also estimated. The work made use of the formation of AuVCs due to the inoculation of Qβ-virus like particles with HAuCl_4_ and NaOH. In the presence of HAuCl_4_ and H_2_O_2_, AuVCs can control the growth of AuNPs depending on the concentration of GSH). The assay implied the deposition of the working solution onto the loading zone of the strip with 2-D computational recognition guiding lines, and the solution would flow through the detection zone, interact with AuNPs and produce deep purple color. When the concentration of GSH increased, AuVCs exhibited decreased peroxidase activity toward H_2_O_2_, leading to the reduced formation of AuNPs and a greyscale value. To obtain the read out, the test strip was placed in an imaging box for capturing the image by smartphone and evaluated GSH concentration by using customized software. The reliability of the LFPB was demonstrated using real cell lines to estimate the correlation between drug-resistance level and intracellular GSH concentration, thereby displaying its potential for onsite clinical diagnosis.

### 3.3. Infectious Diseases

A colorimetric ELISA was improved to enable the detection of *Salmonella enterica Choleraesuis* using urease-induced silver metallization on the surface of gold nanorods (AuNR) [79]. To produce the LSPR change, this strategy involved enzyme-mediated AuNP metallization and silver deposition on nanoparticle surfaces combined with ELISA format. The correlation between urease and *S. enterica Choleraesuis* was monitored via a biotin-streptavidin system. The deposition of silver shell on the surface of AuNRs was induced in the presence of silver nitrate and glucose by the ammonia generated from the hydrolysis of urea after urease capturing. The multicolor change (from blue to brownish yellow) was observed due to the decrease in the aspect ratio (length divided by width) of AuNR, causing a blue shift in the LSPR absorption peak. A detection limit of 1.21 × 10^2^ CFU/mL was obtained for qualitative detection with naked eyes, whereas the quantitative detection was determined at 1.21 × 10^1^ CFU/mL, being two to three orders of magnitude lower than those obtained with conventional horseradish peroxidase HRP-based ELISA. Further analytical parameters such as accuracy and precision were estimated using three different concentrations (1.21 × 10^2^, 1.21 × 10^4^, and 1.21 × 10^6^ CFU/mL). The interference of the matrix as well as the specificity was evaluated by detecting nine common pathogenic bacterial strains, showing good assay selectivity. Although the assay was not applied in clinical samples, the analytical performance was examined in *S. enterica Choleraesuis*-spiked pasteurized whole milk.

**Figure 16:**
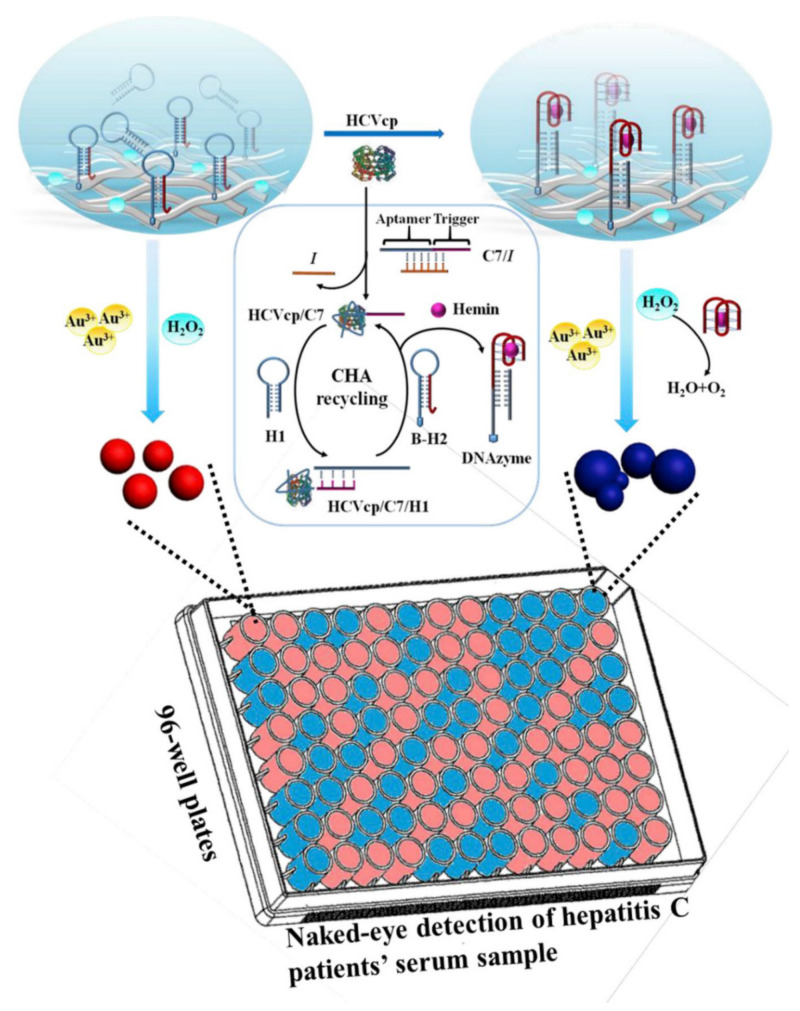
Schematic illustration of the plasmonic nanoplatform for HCVcp detection. Adapted with permission from Li et al. [80] Copyright © 2020 Elsevier.

The detection of hepatitis C virus core protein (HCVcp), an important blood biomarker for the diagnosis of HCV infection in early stages, has been reported using a colorimetric plasmonic nanoplatform [80]. The assay comprised a catalytic hairpin assembly (CHA) amplification reaction combined with polystyrene (PS) nanofibrous membrane and AuNP signal readout (Figure 16). To obtain the visible color shift, the growth of AuNPs was controlled by the functionalization of the nanofibrous membrane. In the presence of HCVcp, the introduction of CHA amplification on the surface of PS continuous film produced a barely detectable change in both the color and the absorbance value at 550 nm. On the contrary, using the PS nanofibrous membrane as the substrate generated a red-to-blue change and a 61% decrease in the 550 nm absorbance value after the HCVcp addition. Under optimized conditions, blue color was observed when the HCVcp concentration was above 10^−4^ pg mL^−1^, whereas HCVcp concentrations below 10^−5^ pg mL^−1^ generated a red color. The analytical practicality was evaluated in whole serum from 30 donors with different viral loads, exhibiting a limit of detection of 1.0 × 10^−4^ pg mL^−1^ by naked eye, which significantly improved ELISA results (LOD: 5 pg mL^−1^). Likewise, the plasmonic nanosensor showed extraordinary potential for POCT due to the good specificity, reusability and long-term stability.

Another plasmonic nanoplatform that uses catalytic hairpin assembly (CHA) amplification efficiency and biocatalytic activity of hemin/G-quadruplex (DNAzyme) is applied to the analysis of Human immunodeficiency virus (HIV) DNA [81]. The growth rate of AuNPs could be controlled by the nanofibrous surface through the enhancement of CHA peroxidase (HRP) and the decomposition catalysis of H_2_O_2_. The naked-eye detection of HIV DNA was also possible in whole serum at 10^−17^ M displaying a red to blue color change. The nanosensor was capable of detecting single- and two-base mismatch at 10^−11^ and 10^−8^ M, respectively. The selective differentiation of single-base mutation reached a discrimination level of 0.001%, while a level of 0.0001% was obtained for two-based mismatch. These features make further development of this application suitable for on-site detection of DNA biomarkers.

A nanosensor platform taking advantage of two configurations of plasmonic core−shell NPs: Au@AgNPs and Ag@AuNPs was developed for the naked-eye biosensing of staphylococcal enterotoxin A (SEA) [82]. The tuning of the optical NPs properties to the cyan-green region was achieved by modifying the silver shell thickness or composition of the complex core−shells. Likewise, the change in the LSPR band to 495 (Ag@AuNPs) and 520 nm (Au@AgNPs) was accomplished by changing the surface chemistry of the shell with anti-SEA antibody (Ab). Consequently, the presence of SEA induced large red shifts of the LSPR band displaying detection limits of 0.2 and 0.4 nM for Au@AgNPs and Ag@AuNPs, respectively. With regard to naked-eye detection, the Ag@AuNPs complex exhibited more distinguishable color variations from orange to red due to the tuning of the initial position of the LSPR band in the cyan to green region (∼500 nm). Since SEA is the most common biotoxin involved in staphylococcal food poisoning outbreaks causing serious consequences on human health, further improvement of this application could be useful for medical diagnostics and environmental monitoring.

A plasmonic ELISA-based assay relied upon the growth of AuNPs for the detection of tuberculosis (TB) analytes in sputum samples with naked-eye [83]. The presence of Mycobacterium tuberculosis ESAT-6-like protein esxB (CFP-10) in the solution was associated with the color change of AuNPs, resulting from the addition of enzyme labels. A color variation from red to blue was observed due to the generation of AuNPs induced by the H_2_O_2_ remaining in solution after the interaction of CFP-10 with catalase labeled antibodies at an absorbance of 550 nm. A limit of detection of 0.01 g mL^−1^ was obtained by the naked eye and the spectrophotometer read out. The testing of CFP-10 in sputum real samples from positive and negatively diagnosed TB patients rendered blue-colored solutions for the positive TB sputum, and red appearance for the negative TB sputum samples. The same reddish color solutions were obtained when examining other proteins (BSA, MPT64), thereby demonstrating the specificity of the plasmonic ELISA sensor for detecting CFP-10.

## 4. Hybrid Biosensing Strategies

Hybrid approaches combining aggregation and non-aggregation sensing mechanisms take advantage of both configurations for enhancing the sensitivity of the assay while achieving ultralow detection of target analytes.

A colorimetric assay comprising the sequential determination of dopamine (DA) and glutathione (GSH) was accomplished using hybrids composed of graphene nanoribbons and silver nanoparticles (GNR/Ag NPs) [84]. The changes in intensity and position of the LSPR absorption band were monitored in the presence of the selected analytes. First, the etching and morphological transition of AgNPs produced a blue shift in the LSPR spectrum of Ag NPs accompanied by a visual color change from green to red due to the addition of DA. Consecutively, the presence of GSH induced the aggregation of nanoparticles in the GNR/Ag NPs-DA mixture, thereby causing a red to gray color change and the decrease in the plasmonic absorption band of Ag NPs. The utilization of the composite formed by GNRs and Ag NPs enabled more sensitive detection of DA and GSH (0.04 and 0.23 mM) than that obtained in the absence of GNRs (0.46 and 1.2 mM), thereby proving the contribution of GNRs in the GNR/Ag NPs hybrid for improving the sensitivity of the assay. The practicality of the colorimetric sensor was successfully applied to the detection of SA and GSH in diluted serum samples showing good recoveries, accuracy and precision.

The identification of protein conformations has also been developed by means of a multidimensional colorimetric sensor array using DNA-AuNPs [85]. This work presents the combined effect of the aggregation and growth of nanoparticles for detecting ten native proteins colorimetrically. The multidimensional capacity of the sensor array was achieved by decorating the AuNPs with a specific oligonucleotide(Apt-5A) or a nonspecific oligonucleotide(16C5A). The color appearance of the solution was modified by both strategies: (i) the aggregation behavior of DNA-AuNPs in the presence of high salt concentrations and (ii) the growth of DNA-AuNPs induced by the reduction of HAuC_l4_ using NH_2_OH as the reductant on their surface and changing their morphology. By using these two receptors, the discrimination of the native proteins and their thermally denatured conformations was accomplished using hierarchical cluster analysis (HCA) at the concentration of 50 nM with 100% accuracy. Additionally, the practical applicability was tested in 50% diluted human urine samples, showing a discrimination accuracy of 36 unknown samples at 500 nM levels. This approach paves the way for the analysis of the conformational changes of biomacromolecules by using the properties of plasmonic nanoparticles for generation of visual detectable color changes.

## 5. Conclusions and Future Perspectives

This work summarizes recent advances in plasmonic colorimetric biosensing on the basis of point of care testing. In terms of practical applicability, colorimetric sensors seem to be ideally suited for promoting personalized medicine in decentralized settings. Among the key characteristics of colorimetric assays, the visual readout is the main advantage over other analytical methods and the most valuable attribute for the monitoring of biological analytes at the point of care.

In the last decade, the development of plasmonic colorimetric sensors has benefited from the fabrication of novel nanomaterials based on either the tuning of their optical properties or the selectivity and stability provided by the chemical functionalization of their reactive surface. The incorporation of various nanomaterials such as metal/oxide nanoparticles, DNA nanozymes or organic probes has been conducted in order to obtain universal and reproducible procedures of synthesis. Likewise, the incorporation of sustainable nanomaterials that support the design of ultrasensitive colorimetric detection configurations is an indispensable requisite for achieving operational devices in the clinical field.

In this sense, the colorimetric analysis of drugs, proteins, DNA, microorganisms and other biological targets has been normally performed by means of aggregation and non-aggregation of nanoparticles strategies depending on inter-particle distance-dependent and size/morphology-dependent principles, respectively. Both approaches are capable of providing visually detectable colorimetric responses by the naked eye without the need for a chromogenic substrate. The colorimetric strategies based on the aggregation of nanoparticles have displayed sensitive and accurate measurements for a large variety of biomolecules by exploiting the coupling of nearby nanoparticles. In spite of potential limitations such as auto-aggregation, the engineering of nanoparticles assemblies is the most common colorimetric sensing scheme due to its simple design. On the other hand, the utilization of non-aggregation procedures has been extensively investigated in recent years to generate distinguishable color changes in the presence of target analytes. By mediating the configuration (size, shape, dielectric environment) of nanomaterials through growth and etching processes, this kind of colorimetric platform normally exhibits higher sensitivities and more control over the analytical performance.

Nevertheless, the great effort devoted to the development of high throughput colorimetric applications has not changed the fact that several issues still remain unanswered. First, the selection of universal plasmonic nanoparticle presentations has not yet been established. Although a wide range of nanomaterials have been explored as colorimetric substrates according to their morphology (spheres, rods, pyramids) or chemical composition (silver, gold), the fabrication of standard materials with the desirable physicochemical stability and biocompatibility has not been entirely addressed. From this point of view, the employment of core–shell nanoparticles combining plasmonic and catalytic activities seems to be a promising way to improve the sensitivity and applicability of colorimetric assays to clinical analysis.

Secondly, the integration of nanoparticles on materials often requires the combination with other technologies to enhance signals via optical, magnetic or electrochemical readers. For instance, the utilization of ELISA and SERS detection in combination with plasmonic-based colorimetric sensing offers the possibility of developing reliable amplification strategies. In contrast, the employment of colorimetric lateral flow immunoassays still demonstrates poor sensitivity and low accuracy in many cases. Subsequently, the enhancement of paper-based microfluidic devices appears to be a feasible alternative to conventional paper test strips as point of care devices. Nevertheless, the number of test strip-based applications in the clinical field is still scarce in comparison with environmental analysis. Similarly, novel signal readout variants involving the incorporation of colorimetric sensing mechanisms into smartphone platforms or wearable devices may contribute to meeting the demand of personalized telemedicine by providing remote monitoring of clinical conditions.

Finally, the application of colorimetric sensing systems to the straightforward analysis of clinical samples mainly relies on the presence of possible interfering compounds in the biological media. However, the nanoparticles behavior not only depends on the interparticle distance or morphological configuration but also on the surrounding dielectric environment. In this regard, most of the colorimetric approaches make use of appropriate sample dilution ratios to avoid the interference of the media composition. Nonetheless, the design of ultralow fouling surfaces is the method of choice for guaranteeing the maintenance of the bioactivity of the sensing surface while ensuring the sensitivity and specificity of the assay. Therefore, the integration of polyethylene and zwitterionic polymers or polysaccharide-based hydrogels as antifouling materials should not be neglected for preventing the undesired effects derived from the composition of the media.

Thus, in spite of the inherent advantages of plasmonic colorimetric sensors over traditional colorimetric systems, more effort is needed to reduce the gap between current proof of concept devices and scalable low-cost platforms in order to achieve the deployment of routine colorimetric-based clinical tests at the point of care.

## Figures and Tables

**Table 1:** Key analytical features of ‘aggregation’-based plasmonic colorimetric sensors classified according to the characteristics of target analyte, plasmonic nanoparticle (namely sensing scheme or biological receptor), color change and limit of detection.

Target Analyte	Plasmonic Nanoparticle/Sensing Scheme/Biological Receptor	Color Change	LOD (Naked-Eye/Spectroscopy) (Biological Sample)	Reference
Protein-related and other biological analytes				
Thrombin	AuNPs modified on 3-aminopropyl-triethoxysilane (APTES)-coated substrates/Dual aptamer	Red to purple	3 mg mL^−1^/1.33 μg mL^−1^ (100-fold—diluted human serum)	[52]
Glucose-6-phosphate dehydrogenase (G6PD) variants	AuNPs segregation in the presence of NADPH (HAuCl4, mediated)	Bluish to red	8.38 mM/Not stated (assay buffer)	[53]
Micro RNA-148a	AuNPs conjugated with oligonucleotides (sandwich hybridization reaction between the conjugated probes and target RNA using “tail-to-tail” or “Head-to-Tail (HT)” alignment)	Red to purple	~1.9 nM (Hybridization buffer)	[54]
Exosomal proteins	AuNPs aggregation in high salt conditions (interaction with a panel of aptamers)	Red to blue	Not stated	[55]
Creatinine	Ag NPs capped with sodium-D gluconate (hydrogen bonds between target analyte and the hydroxyl groups of gluconates)	Yellow to red	0.2 nM (serum 200-fold and urine 1000-fold dilutions times)	[56]
PSA (prostate specific antigen) and CEA (carcinoembryonic antigen)	AuNPs modified with biotin-labeled DNA probe using two enzyme-free and isothermal nucleic acid amplification methods: hybridization chain reaction (HCR) and catalyzed hairpin assembly (CHA)	Red to purple	1 pg mL^−1^ (five human serum samples)	[57]
Cysteine	AuNPs functionalized with β-cyclodextrin (Au-thiol bonds via the thiol group of cysteine molecules)	Wine to red purple	25.47 × 10^−9^ mol dm^−3^ (human urine and serum samples)	[58]
Arginine	Four AuNPs biosynthesized from extract of pomegranate plant (effect of size, shape of the nanoparticles and pH of the medium on the detection of arginine)	Violet to brown (AuNP1); Red to brown (AuNP2); Blue to black (AuNP3) and ash to black (AuNP4)	10^−6^ M range (aqueous solution)	[59]
Clenbuterol, ractopamine	AuNPs functionalized with glutamic acid (Glu) and polyethylenimine (PE) (via NaBH4 reduction method)	Wine red to purple blue	200 nM/0.93–0.98 nM (assay buffer)	[60]
Bacteria and viruses				
SARS-CoV 2	AuNPs capped with suitably designed thiol-modified antisense oligonucleotides (ASOs) specific for N-gene (nucleocapsid phosphoprotein)	Violet to dark blue	0.18 ng mL^−1^ (clinical specimen)	[61]
Urease Bacteria	AuNPs and bovine serum albumin (BSA) induced interaction of positively charged polymer poly-(diallyl dimethylammonium chloride) (PDDA) magnetic beads with negatively charged bacteria or proteins	Red−mauve to gray−blue−violet colors	10^1^ cells mL^−1^ (PBS solution)	[37]
Escherichia coli genomic DNA	AuNP-cluster capturing onto the surface of magnetic microbeads to detect DNA	Pink/red to wine	7.5 × 10^2^ CFU/μL (assay buffer)	[62]
Respiratory Syncytial virus	AuNPs aggregation induced by alkaline phosphatase combined with the loading capacity of magnetic beads and the stimulation effect of zinc ion for signal enhancement/Dual-signal amplified plasmonic ELISA	Gray to red	0.021 pg mL^−1^ (buffer) and 0.035 pg mL^−1^ (spiked serum samples)	[63]
Pharmaceuticals				
Fluoxetine	Silver nanoparticles capped with citrate	Yellow to dark brown	0.18 mg mL^−1^ (human urine and blood serum samples)	[64]
Metformin	Silver nanoparticles modified with cucurbit(6)]uril (CB(6) (in the presence of AgNOs, AgNPs combine with the carbonyl portals of CB(6)	Light yellow to light red	75 μM (50 times diluted urine samples)	[65]
Azithromycin	Silver nanoparticles capped with citrate	Bright yellow to purple	0.2 μM (spiked human plasma)	[66]

LOD: limit of detection; AuNPs: gold nanoparticles; AgNPs: silver nanoparticles.

**Table 2:** Key analytical features of ‘non-aggregation’-based plasmonic colorimetric sensors classified according to the characteristics of target analyte, plasmonic nanoparticle (namely sensing scheme or biological receptor), color change and limit of detection.

Target Analyte	Plasmonic Nanoparticle/Sensing Scheme/Principle	Color Change	LOD (Naked-Eye/Spectroscopy)/(Biological Sample)	Reference
Biological analytes				
Glucose	AuNPs oxidized by glucose oxidase (reduction of HAuCl_4_ by H_2_O_2_)/Growth	Colorless to red	6.28 μM (assay buffer)	[67]
	Gold nanostars (AuNSs) generated by the deposition of silver (oxidation of glucose)/shape altering	Blue to deep purple	0.04–0.12 mmol/L (MES buffer/1000 times diluted serum)	[68]
	Gold nanorods (AuNRs): HRP-H_2_O_2_-3,3′,5,5′-tetramethylbenzidine (TMB) system coupled with an enzymatic reaction to produce H_2_O_2_ in order to etch gold nanorods/Etching	From reddish brown, gray, green, blue, purple, pink to yellow	0.1 to 0.9 mM/1.0–8.0 mM (buffer/ten times diluted human serum)	[69]
	AuNRs: combination of oxidase-catalyzed glucose oxidation and molybdate-catalyzed etching of GNRs by H_2_O_2_	Blue to red to colorless	3 μM/0.45 mM (naked-eye buffer/spiked urine samples)	[70]
	Gold nanobipyramids (AuNBPs): catalysis of horseradish peroxidase (HRP), H_2_O_2_ (generated from glucose oxidation) broken down into hydroxyl radicals (·OH) with strong oxidizability	Green and blue to purple pink	0.02 mM, (100 times diluted serum samples from healthy people and diabetes patients)	[71]
	Au@Ag core-shell NPs involved in situ growth of silver nanoparticles (AgNPs) on the surface of thiol-PEG-capped gold nanoparticles	Orange to red	0.24 mM and 0.15 mM (20 times human urine and serum samples)	[72]
PSA	Silica coated Au@Ag core–shell nanorod (Au@Ag@SiO_2_) Etching/ELISA system(triple read-out including colorimetric, fluorescence and photoacoustic detection)/Dual signal amplification (glucose oxidase (GOx) and magnetic beads)	Green to pink	0.1–1.5 ng mL^−1^ (ELISA/Fluorescence)/(assay buffer)	[73]
	AuNPs coated with ultrathin Platinum/Dual functionality (plasmonic and catalytic activities)/Lateral flow immunoassay	Blue to red	2 ng mL^−1^ (real human serum samples)	[74]
PSA/CEA	AuNRs etching by 3,3′,5,5′-tetramethylbenzidine (TMB), TMB2+/Immunoassay (horseradish peroxidase as the enzyme label and TMB-AuNRs mixture as the chromogenic substrate)	Brown to blue to purple to pink to colorless to yellow	2.5 ng mL^−1^ (PSA) and 75 pg mL^−1^ (CEA) (human serum samples)	[75]
Proteins	AuNRs etching/Oxidization of TMB into TMB^2+^ through H_2_O_2_	Gray to purple to blue to pink	Not stated (identification through hierarchical cluster analysis.)	[76]
Circulating tumor cells	Gold nanorods etching/Peroxidase-like activity of Ethylene diamine tetra acetic acid (EDTA)(decomposition of H_2_O_2_)	Purple to red	7.52 × 10^−15^ U/mL (CA15-3 and PSA: human serum samples)	[77]
Glutathione	AuNPs growth/Gold-viral biomineralized nanoclusters (AuVCs) used as nanozymes/Smartphone auto analysis	Deep purple	9.80 μM (phosphate-buffered saline buffer)	[78]
Bacteria and viruses				
*Salmonella enterica Choleraesuis*	AuNPs enzyme-mediated etching/Silver metallization (urease induced)/ELISA format	Blue to brownish yellow (multicolor)	1.21 × 10^1^–1.21 × 10^2^ CFU/mL CFU/mL (spiked pasteurized whole milk)	[79]
Hepatitis C virus core protein	AuNPs growth/catalytic hairpin assembly (CHA) amplification reaction combined with polystyrene (PS) nanofibrous membrane	Red to blue	1.0 × 10^−4^ pg mL^−1^ (human serum samples)	[80]
Human immunodeficiency virus (HIV) DNA	AuNPs growth/catalytic hairpin assembly (CHA) and biocatalytic activity of hemin/G-quadruplex (DNA zyme)/of CHA peroxidase (HRP) and decomposition catalysis of H_2_O_2_	Red to blue	Not stated/Single- and two-base mismatch at 10^−11^ M and 10^−8^ M, (assay buffer)	[81]
Staphylococcal enterotoxin A (SEA)	Plasmonic core−shell NPs: Au@AgNPs and Ag@AuNPs/Modification of silver shell thickness or composition	Orange to red	0.2 and 0.4 nM for Au@AgNPs and Ag@AuNPs (assay buffer)	[82]
Mycobacterium tuberculosis ESAT-6-like protein esxB (CFP-10)	AuNPs growth/ELISA format (catalase-labeled antibodies, addition of gold (III) chloride, hydrogen peroxide mediated)	Red to blue	0.01 g mL^−1^ (sputum samples)	[83]
Hybrid biosensing strategies				
Dopamine and glutathione	Hybridization of graphene nanoribbons and silver nanoparticles/Etching and Aggregation of AgNPs	Red to gray	0.04 mM (dopamine) and 0.23 mM (glutathione) (5–100-fold diluted human serum samples)	[84]
Protein conformations	AuNPs decorated with specific and nonspecific oligonucleotides/Aggregation (salt) and Growth (HAuC_l4_ using NH_2_OH)	Purple to red to blue	50 nM (50% diluted urine samples)	[85]

LOD: limit of detection; AuNPs: gold nanoparticles; AgNPs: silver nanoparticles.
